# A Comparative Study on Microstructure and Mechanical Properties of Ti-6Al-4V Fabricated by Laser/Electron Beam Powder Bed Fusion

**DOI:** 10.3390/ma19153300

**Published:** 2026-08-04

**Authors:** Yaojia Ren, Jingru Wang, Jiajun Xu, Yingkang Wei, Jilei Zhu, Qingge Wang, Jianyong Wang, Shifeng Liu, Solomon-Oshioke Agbedor

**Affiliations:** 1 School of Metallurgical Engineering, Xi’an University of Architecture and Technology, Xi’an 710055, China; 125ren@163.com (Y.R.); wjr112036@163.com (J.W.); 13571532811@163.com (J.X.); weiyingkang@xauat.edu.cn (Y.W.); zhu_jl@126.com (J.Z.); wendymewqg@163.com (Q.W.); wangjy@xauat.edu.cn (J.W.); 2 State Key Laboratory of Powder Metallurgy, Central South University, Changsha 410083, China; solomon.agbedor@yahoo.com

**Keywords:** additive manufacturing, titanium, microstructure, mechanical properties

## Abstract

To address the strength–ductility trade-off in titanium alloys, a comparative study was conducted on Ti-6Al-4V (TC4) alloys fabricated by laser powder bed fusion (L-PBF) and electron beam powder bed fusion (EB-PBF). The L-PBF specimen primarily consisted of acicular α′ martensite with high residual stress. In contrast, the EB-PBF specimens, owing to a substrate preheating temperature of 740 °C and a reduced cooling rate (10^3^~10^5^ K/s), exhibited a stable and coarse α + β lamellar structure. Combined with the high oxygen content (0.24 wt.%) that provided solid-solution strengthening, this morphology enabled simultaneous attainment of a yield strength of 1120 ± 12 MPa and an elongation at fracture of 11.1 ± 1.3%. Notably, deformation-induced HCP→FCC phase transformation occurred in EB-PBF alloys, generating a dual-phase HCP/FCC structure that effectively accommodated plastic strain. These results highlight the superior potential of EB-PBF over L-PBF for fabricating titanium alloys with an exceptional strength–ductility synergy.

## 1. Introduction

Titanium alloys, developed since the 1950s, have become indispensable structural materials across demanding industries owing to their exceptional property combinations, including high specific strength, corrosion resistance, and biocompatibility. Among these alloys, Ti-6Al-4V (TC4) is the most widely used titanium alloy worldwide because of its excellent mechanical properties and versatility in the aerospace, medical, and automotive sectors [1,2,3,4,5]. However, conventional manufacturing routes such as forging and casting face inherent limitations: low material utilization (often below 15%), high production costs, and restricted design freedom. These constraints impede the fabrication of components with complex geometries or customized architectures, limiting their adaptability to modern engineering demands. Additive manufacturing (AM) has emerged as a transformative solution by enabling layer-by-layer deposition. This approach achieves near-net-shape production with minimal material waste, shortened processing cycles, and unprecedented geometric flexibility [6,7]. It has revolutionized the production of components previously impossible or economically unfeasible via traditional methods like machining or casting. Despite challenges such as inherent porosity, residual stresses, anisotropic mechanical properties, and, in some cases, inferior fatigue performance compared with conventionally processed alloys [8], the technique enables the fabrication of intricate internal channels, topology-optimized lightweight structures, and patient-specific implants. These capabilities position additive manufacturing as a disruptive manufacturing technology for industries that prioritize customization, weight reduction, and performance optimization.

Among the various AM techniques, laser powder bed fusion (L-PBF) and electron beam powder bed fusion (EB-PBF) are prominent for processing titanium alloys. Both belong to the powder bed fusion category and enable near-net-shape fabrication of geometrically complex components with high dimensional accuracy; moreover, for such AM titanium components, post-treatment techniques such as reactive electro-spark deposition have been reported to improve surface quality, hardness, and wear resistance [9]. They differ fundamentally in energy sources, process environments, thermal profiles, and microstructural outcomes [10,11]. L-PBF operates in an inert argon/nitrogen atmosphere using a high-power laser beam to selectively melt successive layers of metal powder, inducing rapid cooling rates (10^4^–10^6^ K/s). These conditions often lead to as-built specimens exhibiting high strength but low ductility (≤10%). For instance, Kumar et al. [12] reported that the as-built TC4 alloy, containing α/α′ phases, achieved a yield strength of 1161 MPa but only 7.6% elongation. It is widely accepted that the low ductility of L-PBF-processed titanium alloys stems from the formation of α′ martensite [13,14,15].

To address this, post-heat treatments are commonly applied to convert the metastable α/α′ phase into a stable α + β dual-phase structure, thereby achieving a balance between strength and ductility. Waqas et al. [16] demonstrated that solution treatment at 950 °C followed by a 4 h aging at 500 °C produced a TC4 alloy with a yield strength of 932 MPa and an elongation of 15.6%. Nevertheless, such use of heat treatment increases process complexity and cost, driving interest in alternative approaches to achieve optimal mechanical properties directly through process control. Recent work by Xu et al. [14] demonstrated that controlling the inter-layer time enables in situ decomposition of α′ martensite through thermal cycling. Applying this strategy, they successfully fabricated a TC4 specimen with a lamellar (α + β) structure, achieving a yield strength of 1022 MPa and an elongation of 12.7%. Owing to the unique thermal history in AM, the top region of the specimen often retains α′ martensite as a result of insufficient heat accumulation [14,17]. Some studies further suggest that the poor ductility is not attributed to the presence of α′, but arises from the significant stress concentration induced at the α′/β interface during deformation, particularly when thin β lamellae are present [18,19,20]. In light of this, Voisin et al. [21] produced a fully α′ TC4 alloy that exhibited an elongation higher than 10%.

In contrast, EB-PBF is conducted in a high-vacuum environment, where a build platform preheated to approximately 700 °C is utilized, and each layer of powder is systematically pre-sintered using an electron beam prior to melting [11]. The preheating temperature above 700 °C promotes the complete decomposition of the α′ phase within one hour. Owing to its relatively low cooling rates (10^3^~10^5^ K/s) [22], the formation of coarse grains is promoted, resulting in TC4 alloys with reduced strength but enhanced ductility. For instance, Tan et al. [23] found that EB-PBF TC4 specimens possess a coarse lamellar (α + β) structure with a high elongation of 16.4%, while the yield strength was only 852 MPa. Bi et al. [24] further demonstrated that EB-PBF TC4 specimens exhibited minimal anisotropy between the horizontal and vertical directions, with a yield strength of ~881 MPa and an elongation of ~13.6%. This indicates that the process effectively mitigates the influence of thermal cycling on directional sensitivity, reflecting a high degree of microstructural homogeneity. Additionally, Tang et al. [25] investigated the effect of powder reuse on the properties of EB-PBF TC4. As the number of powder recycling cycles increased, the oxygen content rose from 0.08 wt.% to 0.19 wt.%, the yield strength increased to 960 MPa, and the elongation was retained at 15.5% despite a minor reduction.

Despite these advancements, a fundamental understanding of how distinct interface types govern tensile properties remains elusive. Specifically, the differential impact of the acicular α′/α′ boundaries in L-PBF versus the lamellar α/β interfaces in EB-PBF on strain accommodation has not been systematically elucidated. While L-PBF struggles with the ductility limit imposed by brittle α′ martensite, EB-PBF faces the challenge of lower strength due to grain coarsening. Importantly, the role of interstitial oxygen in EB-PBF is often oversimplified as a defect causing embrittlement. The potential for utilizing oxygen uptake to tailor the lattice resistance and activate novel strengthening mechanisms within the α + β structure, thereby achieving a synergy between strength and ductility, represents a critical yet underexplored pathway for microstructural design.

Therefore, in this work, the microstructural evolution and deformation behavior of TC4 alloys produced by two prominent AM techniques are systematically examined, with emphasis on the influence of processing conditions on phase formation, grain morphology, and mechanical performance. A key innovation of this study is the introduction of an elevated oxygen content into EB-PBF TC4 to achieve the strength–ductility synergy. By elucidating the role of oxygen in governing the microstructure and mechanical response of EB-PBF TC4, this study provides guidance for optimizing additive manufacturing strategies to achieve a better balance between strength and ductility.

## 2. Experiment

TC4 specimens were fabricated using L-PBF and EB-PBF techniques. For L-PBF, TC4 powder with a particle size range of 15–53 μm was utilized to produce specimens measuring 50 mm × 40 mm × 30 mm on a BLT-S210 system (Bright Laser Technologies, Xi’an, China). The process was conducted under an inert argon atmosphere with the following parameters: a substrate preheating temperature of 200 °C, a laser power of 300 W, a scanning speed of 1029 mm/s, a hatch spacing of 0.12 mm, a layer thickness of 0.06 mm, a focal offset of 2 mm, and a 67° rotation scanning strategy applied between successive layers. EB-PBF processing employed recycled TC4 powder from previous build cycles with a particle size range of 54–105 μm. Manufacturing was performed using an S-200 system (Sailong, Xi’an, China) under high-vacuum conditions. A commercially pure titanium (CP-Ti) platform was used as the substrate. The process parameters included a substrate preheating temperature of 740 °C, an accelerating voltage of 60 kV, a beam current of 18 mA, a scanning speed of 5400 mm/s, a beam spot diameter of 0.10 mm, and a layer thickness of 0.05 mm. All parameter combinations were validated through preliminary printing trials, ensuring that the relative densities of both L-PBF and EB-PBF specimens reached approximately 98%, meeting the basic quality requirements for subsequent tensile testing and microstructural characterization.

After fabrication, the dog-bone tensile specimens were machined from the bulk specimens using electrical discharge wire cutting. Relative density was measured by the Archimedes method with a Densimeter (MS-DNY-43, Shenyang, China). Phase identification was carried out by X-ray diffraction (XRD) using a D8 Advance diffractometer (Bruker, Karlsruhe, Germany) with Cu-*K*_α_ radiation (λ = 0.15406 nm) over a 2*θ* range of 30–80° and a step size of 0.02°. Oxygen content measurements conducted using an oxygen–nitrogen–hydrogen analyzer (ONH-2000, Eltra, Haan, Germany) revealed values of 0.12 wt.% and 0.24 wt.% for the L-PBF and EB-PBF specimens, respectively. The specimens for optical microscopy (OM) were polished using standard metallographic techniques followed by electropolishing. For microstructural examination, the specimens were etched with Kroll’s reagent (2% HF + 6% HNO_3_ + 92% H_2_O) for 15–20 s. Scanning electron microscope (SEM) observations and electron backscatter diffraction (EBSD) analyses were performed using a JSM-7200F (JEOL, Akishima, Japan). The foils were mechanically ground to approximately 30 μm in thickness, thinned by Ar-ion milling, and examined using a Talos F200X transmission electron microscope (TEM, FEI, Hillsboro, OR, USA). Tensile tests were conducted at room temperature with an AGS-X universal testing machine (Shimadzu, Kyoto, Japan) at a strain rate of 10^−3^ s^−1^, using a non-contact digital video extensometer for strain measurement. At least three tensile specimens were tested for each condition to ensure experimental reproducibility.

## 3. Results

**Figure 1:**
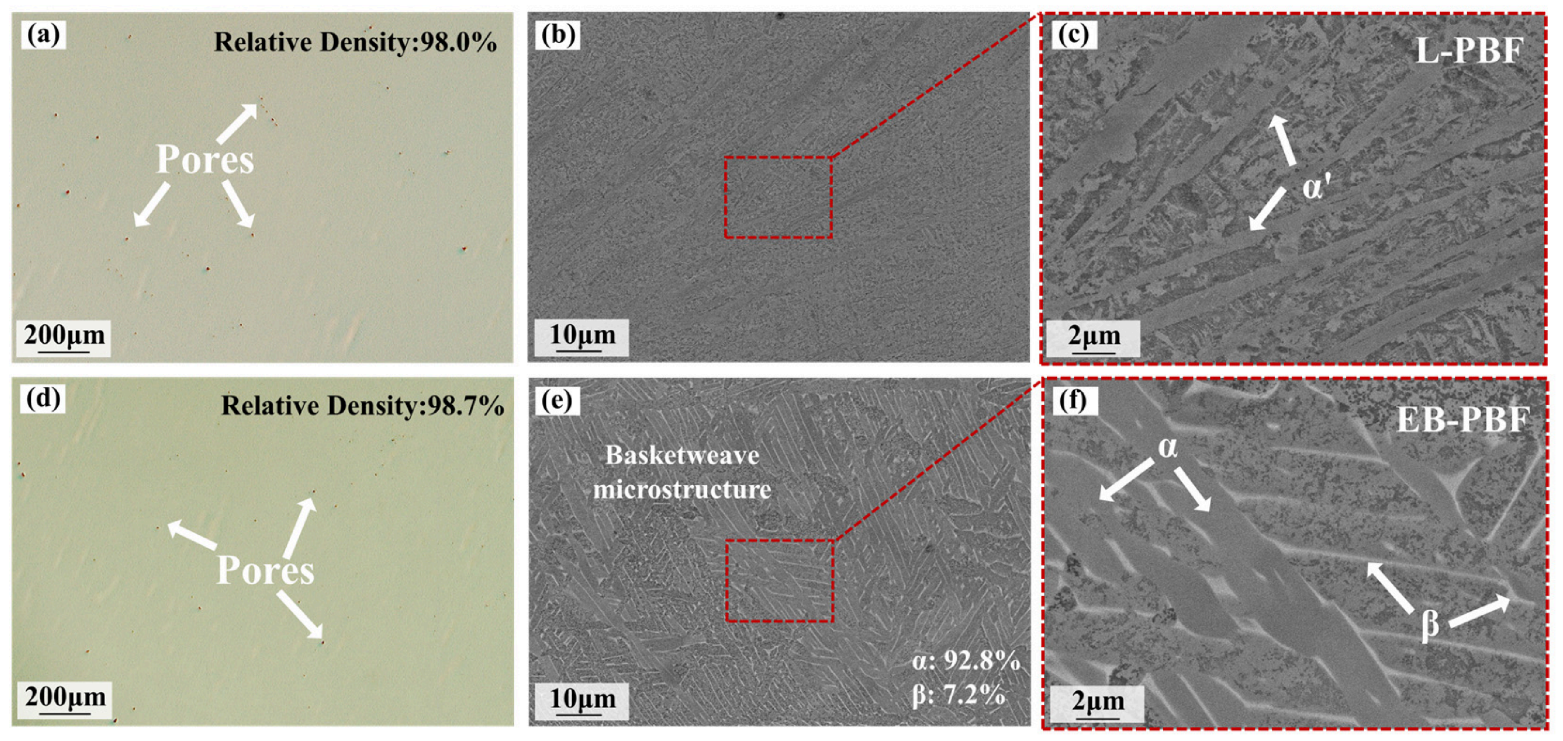
(**a**,**d**) OM and (**b**,**c**,**e**,**f**) backscattered electron (BSE) images of (**a**–**c**) L-PBF and (**d**–**f**) EB-PBF specimens.

The relative densities of the L-PBF and EB-PBF specimens, as measured by Archimedes’ displacement, were 98.0 ± 0.7% and 98.7 ± 0.4%, respectively. Optical micrographs in Figure 1a,d display polished surfaces with visible pores and calculated porosity percentages matching Archimedean relative density values. The BSE image in Figure 1b shows the L-PBF microstructure dominated by α′, resulting from the inherent extremely high cooling rate. The enlarged view in Figure 1c highlights the ultrafine martensitic laths, which exhibit an average thickness of approximately 0.7 μm. Figure 1e reveals the EB-PBF microstructure with a characteristic basket-weave morphology, where ImageJ 1.54p quantification identified 92.8% α-phase and 7.2% β-phase fractions. Figure 1f, an enlarged view corresponding to the dashed square in Figure 1e, reveals coarser α laths (~1.9 μm thickness) with a retained V-enriched β phase [26].

**Figure 2:**
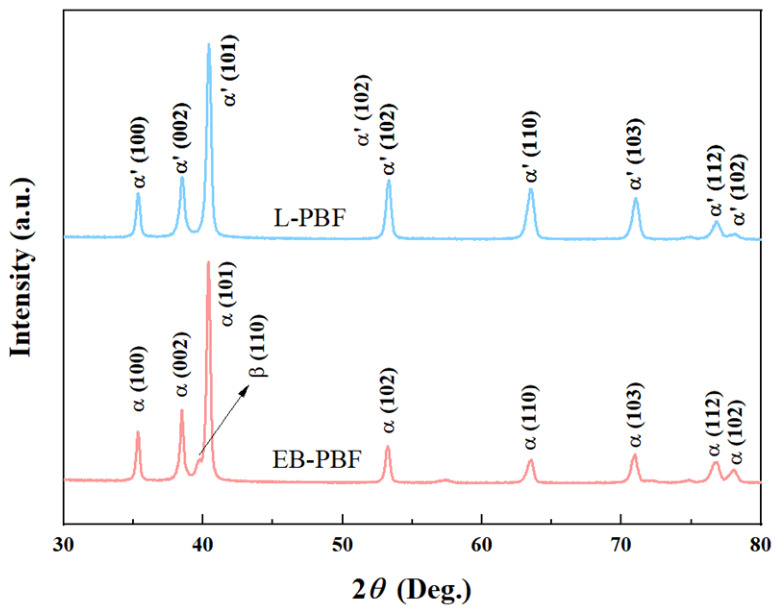
XRD patterns of the L-PBF and EB-PBF specimens.

The XRD patterns for both specimens are depicted in Figure 2. In the L-PBF specimen, only α′ peaks are detected, which is attributed to the suppression of β-phase retention caused by the extremely high cooling rate. In contrast, the EB-PBF specimen exhibits minor β-(110) and β-(200) diffraction peaks, indicating that β-phase stability is enhanced by the higher preheating temperature (740 °C) and the moderate cooling rate. These thermal conditions are shown not only to stabilize the β-phase but also to completely inhibit the formation of residual α′ martensite, resulting in a thermodynamically stable α + β dual-phase structure. Additionally, noticeable peak broadening is observed in the α′ reflections of the L-PBF specimen.

**Figure 3:**
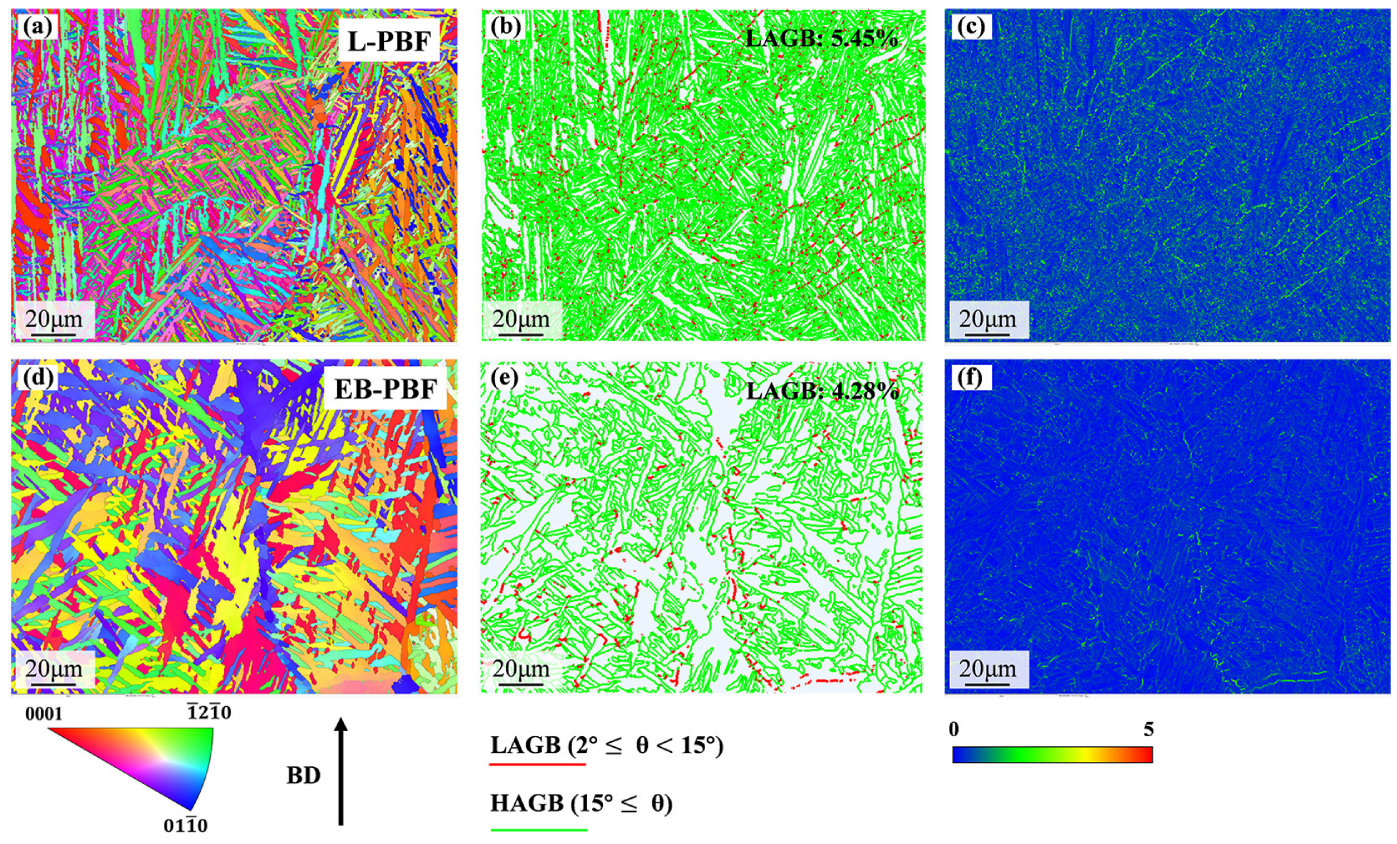
(**a**,**d**) IPF maps, (**b**,**e**) grain-boundary misorientation maps, and (**c**,**f**) KAM maps of the L-PBF and EB-PBF specimens.

To further elucidate the process-induced microstructural differences, EBSD analyses were performed on the as-built specimens. Inverse pole figures (IPFs) of the as-built specimens are presented in Figure 3a,d. Solidification in both cases initiates from a single parent β grain; the α variants formed within prior β grains exhibit limited orientation relationships. The EB-PBF process involves a higher substrate preheating temperature and reduced cooling rate, promoting the development of a more consistent crystallographic orientation in the α-phase. This behavior results from slower cooling and prolonged thermal exposure, providing favorable conditions for α-lath growth. The process facilitates the precipitation of α variants with closely aligned orientations, forming lamellar and clustered structures, and significantly enhancing texture intensity.

Grain-boundary misorientation maps for the as-built specimens are shown in Figure 3b,e. Red lines indicate low-angle grain boundaries (LAGBs, 2–15°), while green lines correspond to high-angle grain boundaries (HAGBs, ≥15°). Consistent with the IPF analysis, the EB-PBF specimen exhibits a lower fraction of LAGBs (4.28%) compared with the L-PBF specimen (5.45%). This reduction can be attributed to the slower cooling rate and elevated substrate preheating temperature in EB-PBF, which promote subgrain coalescence and the elimination of low-angle boundaries.

The kernel average misorientation (KAM) map reflects the average misorientation between the grain center orientation and its nearest neighbors, and therefore, the KAM maps can be used to reveal the distribution of local strain. The color scale below the KAM maps indicates the misorientation degree, where blue (0°) corresponds to minimal misorientation and red (5°) represents the maximum value. As shown in Figure 3c,f, the L-PBF specimen exhibits higher local misorientation compared to the EB-PBF specimen, which is attributed to the higher cooling rate of L-PBF. This results in greater residual stress, thereby increasing the likelihood of crack formation and other defects. These differences mainly originate from the distinct cooling rates and thermal histories associated with L-PBF and EB-PBF processing.

**Figure 4:**
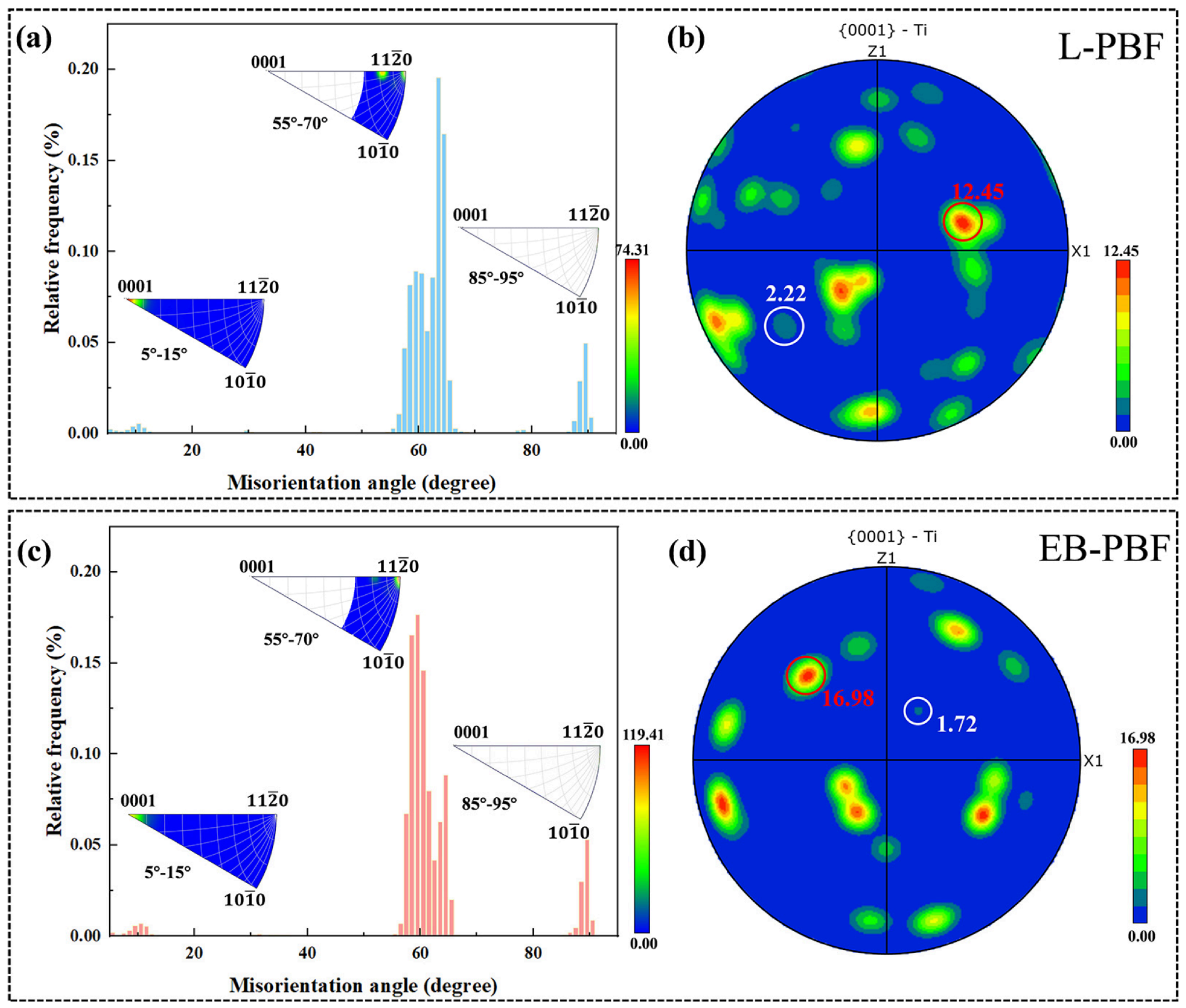
(**a**,**c**) Misorientation angle distributions and (**b**,**d**) PFs of the L-PBF and EB-PBF specimens; the insets show the misorientation axes for angle ranges of 5–15°, 55–70°, and 85–95°.

Systematic characterization was conducted for the misorientation angles (>5° only) between the neighboring α-variants in the specimens under all processing conditions, with the results summarized in Figure 4a,c. The three IPF insets in each plot display the misorientation axes corresponding to misorientation angle ranges of 5–15°, 55–70°, and 85–95°, respectively. Significant differences in misorientation distribution were observed between the two processes. In L-PBF specimens, the Type 4 α/α boundary with a characteristic misorientation angle of ~63° is prevalent. In contrast, EB-PBF specimens were characterized by a higher frequency of Type 2 α/α boundaries, exhibiting a misorientation angle of 60° [27].

The {0001} pole figures (PFs) of the α-phase in the specimens under both processing conditions are presented in Figure 4b,d. When all α-variants form randomly, the intensity of all {0001} poles is expected to be uniform, with a pole intensity ratio close to 1.0. A maximum-to-minimum pole intensity ratio (indicated by red and white circles, respectively) significantly greater than 1.0 indicates the occurrence of variant selection. This ratio was found to be substantially higher than 1.0 in the specimens from both processes, confirming that variant selection took place. The maximum multiple of uniform distribution (MUD) value in the specimen under EB-PBF processing (16.98) exceeded that in the specimen under L-PBF processing (12.45), indicating a stronger variant selection tendency and more pronounced texture intensity in the specimen under EB-PBF processing.

**Figure 5:**
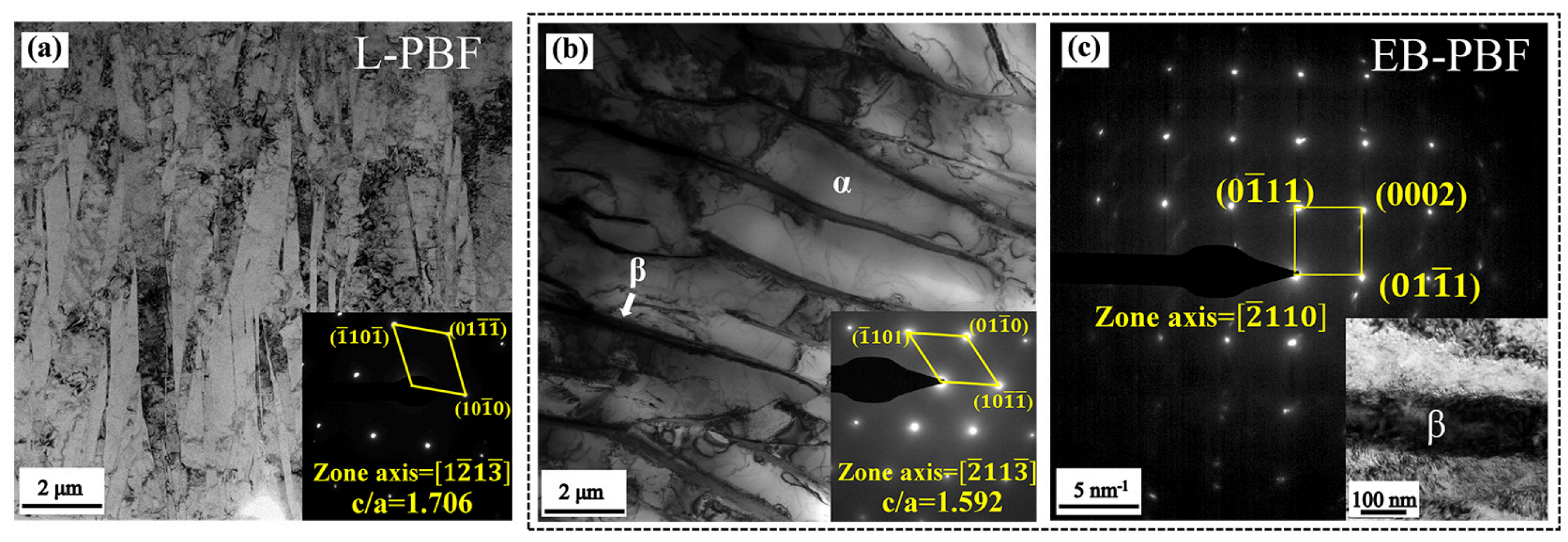
BF images of the (**a**) L-PBF and (**b**) EB-PBF specimens; the insets show the SAED patterns corresponding to the HCP structure. (**c**) SAED pattern of the BCC structure; the inset is an enlarged view of the BCC lamellar.

Figure 5a presents a bright-field (BF) image of the L-PBF specimen, characterized by ultrafine α′ martensitic laths. The SAED pattern in the inset, indexed along the [$1\bar{2}1\bar{3}$] zone axis, confirms an HCP structure. It is clear that the measured c/a ratio of 1.706 deviates from the ideal value of 1.586, indicating lattice distortion, which in turn signifies the presence of the α′ martensitic matrix. Figure 5b shows a BF image of the EB-PBF specimen, revealing coarser α-laths coexisting with the β-phase. The SAED pattern in the inset, obtained along the [$1\bar{2}1\bar{3}$] zone axis, shows a c/a ratio of 1.592, closely matching the theoretical HCP value of 1.586 and indicating minimal lattice distortion. This is attributed to the low cooling rate and high preheating temperature in EB-PBF, which allow sufficient atomic diffusion and promote the formation of a stable α-phase. The SAED pattern in Figure 5c, indexed along the [$\bar{1}00$] zone axis of a BCC structure, provides direct evidence of retained β-phase. These observations are consistent with the BSE and XRD analyses, further confirming that the EB-PBF specimens consist of lamellar (α + β) structures.

**Figure 6:**
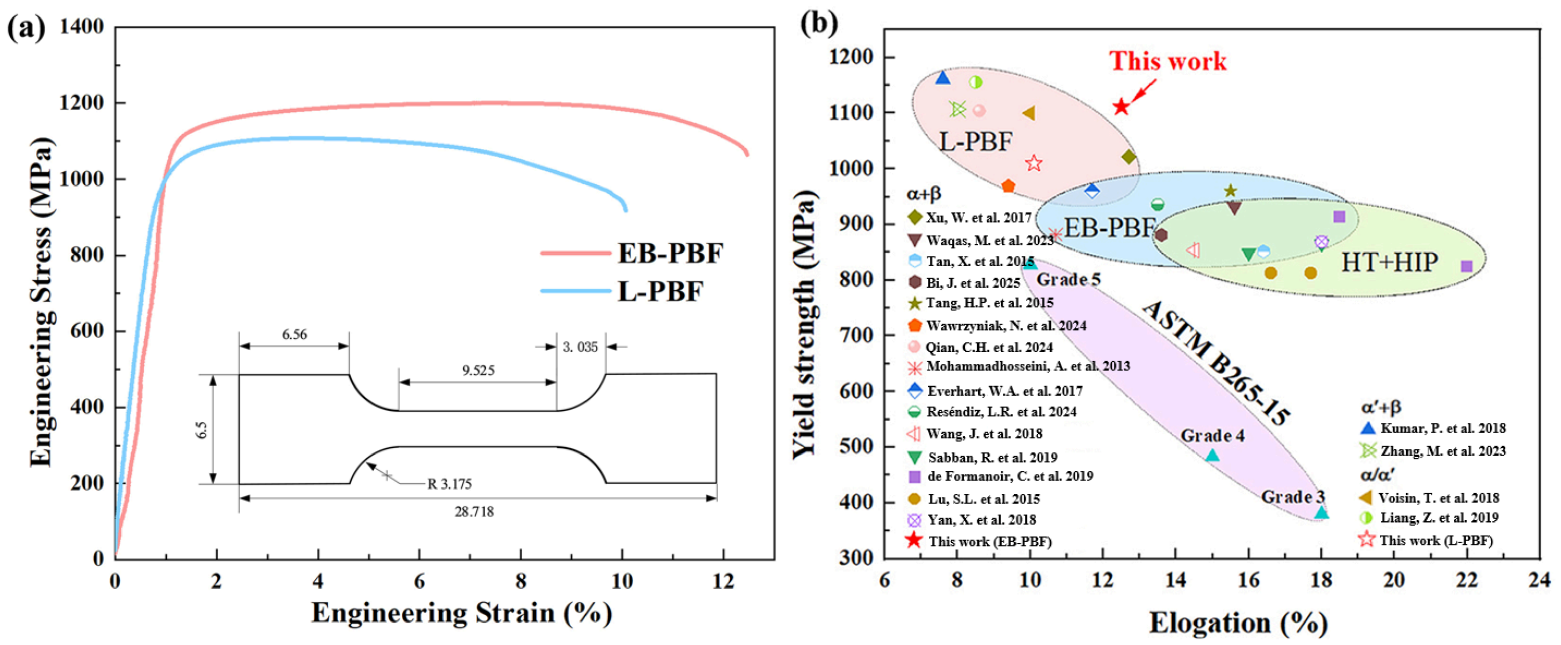
(**a**) Engineering stress–strain curves of the L-PBF and EB-PBF specimens, (**b**) yield strength and elongation to failure of TC4 alloys in the references reported so far [12,14,16,21,23,24,25,28,29,30,31,32,33,34,35,36,37,38,39].

The tensile engineering stress–strain curves of the as-built specimens are shown in Figure 6a. The EB-PBF specimens exhibit superior mechanical performance, with a yield strength of 1120 ± 12 MPa and an ultimate tensile strength of 1209 ± 25 MPa, both of which are higher than those of the L-PBF specimens (1016 ± 32 MPa and 1104 ± 20 MPa, respectively). Comparable elongation is maintained, with values of 11.1 ± 1.3% for EB-PBF and 10.0 ± 0.9% for L-PBF. Notably, the uniform elongation of the L-PBF specimens was only ~3.8 ± 0.1%, while that of the specimen under EB-PBF processing increased by 78.9% to 6.8 ± 0.3% (Figure S1). A summary of tensile properties from previous research is provided in Figure 6b [12,14,16,21,23,24,25,28,29,30,31,32,33,34,35,36,37,38,39], demonstrating that the EB-PBF TC4 specimens in this work achieve an excellent combination of strength and ductility.

**Figure 7:**
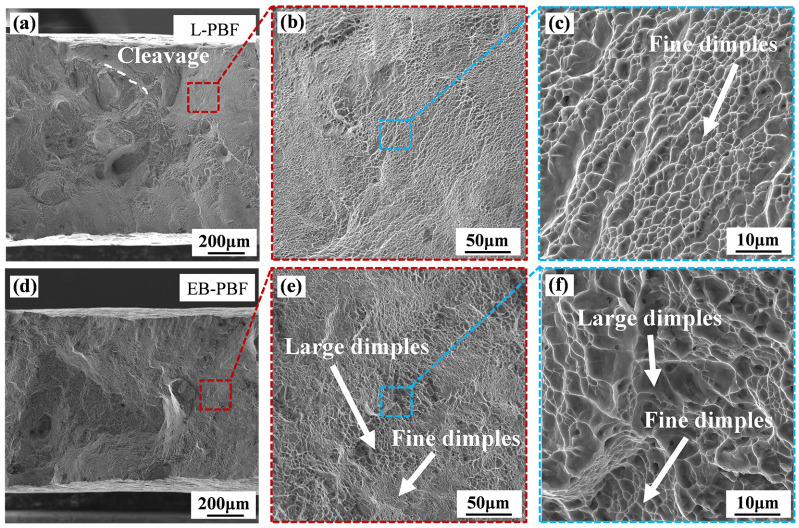
Fractography of the (**a**–**c**) L-PBF and (**d**–**f**) EB-PBF specimens; (**b**,**e**) and (**c**,**f**) are the enlarged views taken from the dashed square regions in (**a**,**d**) and (**b**,**e**), respectively.

Fractographic analyses revealed distinct differences in the fracture characteristics between the L-PBF and EB-PBF specimens, as shown in Figure 7. Figure 7a–c show the fracture surface of the L-PBF specimens, which exhibit a mixed morphology consisting predominantly of fine, shallow dimples, along with localized quasi-cleavage features. This morphology suggests limited microvoid coalescence and restricted plastic deformation during fracture. In contrast, the EB-PBF specimen demonstrates a typical ductile fracture mode characterized by a bimodal dimple structure comprising both large and small dimples, as shown in Figure 7e,f. The presence of deep and well-developed dimples reflects enhanced plastic deformation capability, which is consistent with the superior ductility measured in the tensile tests.

**Figure 8:**
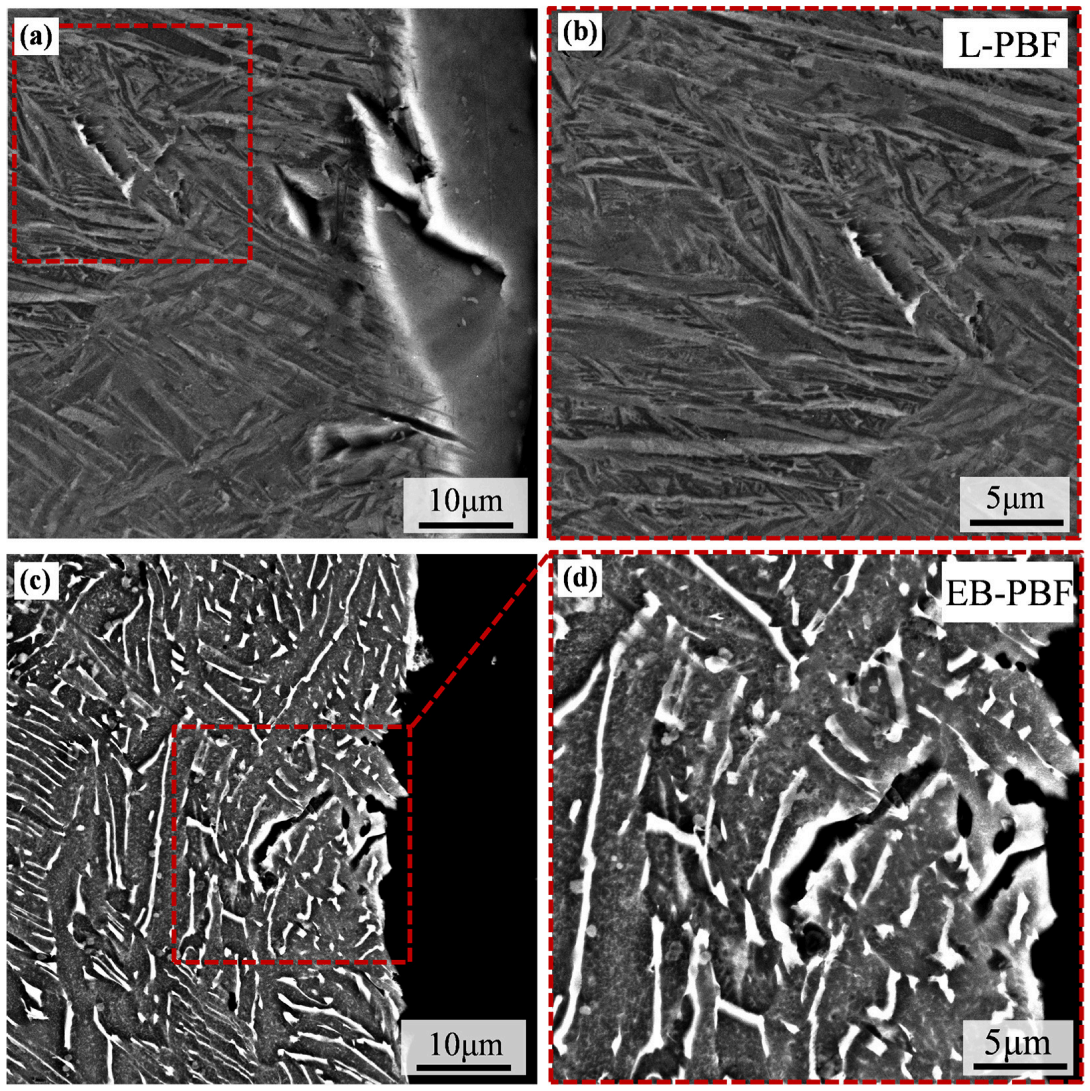
BSE images of the (**a**,**b**) L-PBF and (**c**,**d**) EB-PBF specimens; (**b**) and (**d**) are the enlarged views taken from the dashed square regions in (**a**) and (**c**), respectively.

As shown in Figure 8a,b, the microstructure of the L-PBF specimen is dominated by elongated α′ martensite laths. The extremely high cooling rate of this process results in significant local residual stresses, with pronounced stress concentration particularly distributed along the lath boundaries. Consequently, microcracks tend to initiate and propagate along these long and straight interfaces, leading to predominantly linear crack paths. In contrast, the EB-PBF specimen (Figure 8c,d) exhibits a microstructure characterized by thicker and shorter α laths with approximately 7.2% retained β phase. This morphological difference stems from the relatively lower cooling rate and the preheating of the substrate to 740 °C, which effectively reduces thermal gradients and promotes the decomposition of the α′ phase. Within this microstructure, microcracks are deflected upon encountering short lath boundaries, often propagating along one interface for a certain distance before transitioning to an adjacent one, resulting in curved crack propagation patterns. Furthermore, the retained β phase in the EB-PBF specimen demonstrates excellent plastic compatibility, enabling local stress relaxation through plastic deformation [40], thereby delaying crack propagation. To quantitatively characterize crack propagation behavior, ImageJ software was utilized to perform statistical analysis on at least three crack paths for each group of specimens [41]. The average crack tortuosity factor of the EB-PBF specimens is 1.23 ± 0.01, compared with 1.02 ± 0.02 for the L-PBF counterparts. Such quantitative indices firmly confirm that the lamellar α + β microstructure significantly facilitates crack deflection, which is one of the core reasons for the superior ductility of EB-PBF Ti-6Al-4V. It is therefore concluded that the curved crack paths, the strain accommodation capacity provided by the β phase, and the relatively lower initial residual stress collectively govern the fracture behavior of EB-PBF specimens.

**Figure 9:**
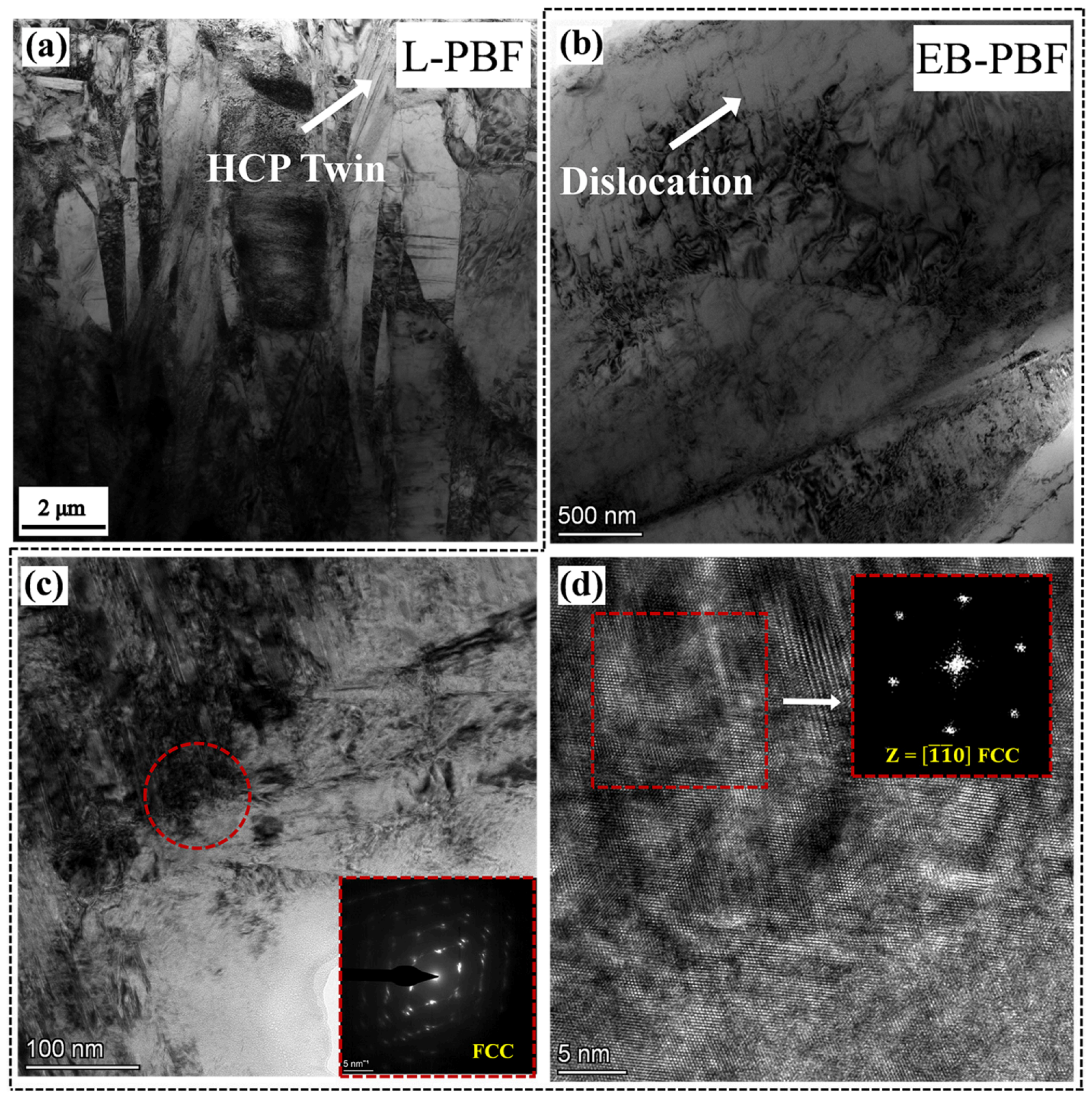
BF images of the (**a**) L-PBF and (**b**,**c**) EB-PBF specimens; (**d**) HRTEM image of the ultrafine lath, the inset is the FFT pattern taken from the dashed square, showing the FCC phase.

The mechanical properties, fracture morphologies, and crack propagation behaviors of the specimens produced by the two processes exhibit significant differences. To elucidate the strengthening mechanisms of the respective processes, TEM characterization was performed on the deformed specimens, as shown in Figure 9a. Plastic deformation in the L-PBF TC4 alloy is accommodated through the formation of HCP twins within the matrix. The formation of such nanotwinning structures is primarily attributed to the inherent high residual stresses and thermal cycling-induced atomic rearrangements during the L-PBF process, a phenomenon that has also been well-documented in previous studies [42,43,44]. Additionally, it is revealed in Figure 9b that a high density of dislocations is accumulated and tangled at the α/β phase interfaces, leading to the formation of a significant local stress concentration. It is further observed that this dislocation configuration effectively hinders the further glide of subsequent dislocations, resulting in the enhancement of the material’s yield strength through dislocation strengthening. A bright-field TEM image is provided in Figure 9c, in which the presence of a region with ultrafine laths can be identified in the upper-right area. The SAED pattern acquired from the red-circled region is shown in the inset. Several sets of overlapping diffraction spots can be observed and tentatively indexed as FCC Ti. These overlapping reflections may originate from FCC nanodomains with different crystallographic orientations. To further verify the phase assignment and minimize ambiguity arising from the overlapping diffraction signals, high-resolution characterization was performed in a representative local region, as shown in Figure 9d. The fast Fourier transform (FFT) pattern inset, corresponding to the red-boxed area in Figure 9d, was indexed along the zone axis [$\bar{1}\bar{1}$0] and exhibited diffraction features consistent with an FCC structure, thereby providing further support for the presence of the FCC phase.

## 4. Discussion

### 4.1. Phase Transformation During the L-PBF and EB-PBF Processes

In AM of titanium alloys, the distinct thermal histories inherent to L-PBF and EB-PBF approaches give rise to markedly different phase constitutions and microstructures. Conventional TC4 alloys typically exhibit equiaxed grains with a near-equilibrium α + β dual-phase microstructure. However, the extremely high cooling rate of L-PBF (10^4^~10^6^ K/s) significantly suppresses diffusional phase transformations. This drives a direct β→α′ diffusionless martensitic transformation, resulting in a fine, nonequilibrium microstructure composed of α′ martensite with an average thickness of ~0.7 μm. Importantly, a pronounced thermal gradient develops along the build direction (BD) during L-PBF: at the bottom region, repeated thermal cycling induces in situ decomposition of α′, whereas at the top, insufficient thermal input preserves α′, thereby giving rise to significant differences in both microstructure and mechanical properties [45]. It is challenging to completely eliminate α′ in the top region of the L-PBF specimen. Therefore, in this study, β was transformed, rather than relying on in situ decomposition of α′ martensite, to obtain a specimen composed entirely of α′ martensite.

The EB-PBF process benefits from its high-vacuum environment and elevated substrate preheating, which together establish a relatively stable thermal field and thereby provide a pronounced advantage in phase transformation control. During cooling, EB-PBF specimens predominantly undergo the β → α + β transformation, where the β-phase directly transforms into equilibrium α rather than α′ [23,46]. This behavior is primarily attributed to the high preheating temperature exceeding the martensitic start temperature, effectively suppressing the diffusionless transformation. As a consequence, the resulting microstructure exhibits relatively coarse α laths (~1.9 μm) while retaining a minor fraction of β-phase (~7.2%), yielding a more stable α + β dual-phase structure. Analysis of variant selection and PFs revealed that EB-PBF specimens had a high fraction of Type 2 α/α boundaries, indicative of a relatively low cooling rate and the prevalence of equiaxed prior-β grains. This observation is consistent with Lu et al. [47], who showed that reducing the cooling rate in L-DED via an ultrasonic-assisted external field favored the formation of Type 2 α/α variants within equiaxed grains.

SAED analysis further highlights differences in lattice stability between the two specimens. The higher c/a ratio observed in the L-PBF specimens compared to the EB-PBF specimens suggests that the retention of the α′ metastable phase is primarily due to kinetic constraints imposed by rapid solidification, rather than thermodynamic preference. In contrast, the formation of the FCC phase requires the simultaneous satisfaction of two conditions: stringent thermodynamic prerequisites and adequate stress levels. This explains the lack of the FCC phase in the L-PBF specimens, consistent with previous reports describing a gradient distribution of FCC phases along the BD. This can be attributed to the relatively low substrate preheating temperature in L-PBF (approximately 200 °C), where the thermal accumulation along the BD is insufficient to meet the thermodynamic conditions required for FCC formation, which typically demands temperatures exceeding 600 °C [48]. Although EB-PBF provides a thermal environment with a substrate preheating temperature of 740 °C capable of meeting the thermodynamic criteria for FCC formation, the stresses experienced during printing remain below the critical threshold for in situ FCC phase nucleation.

EB-PBF utilizes a high-vacuum environment and the steady-state thermal field activated by a preheated substrate at 740 °C to develop a near-equilibrium microstructure. This approach fundamentally addresses the phase-gradient distribution and residual stress concentration issues commonly encountered in L-PBF processing. The primary α-phase in the EB-PBF specimen possesses a nearly ideal HCP structure with negligible distortion, which substantially reduces the energy barrier for the subsequent stress-induced HCP → FCC transformation. The improved lattice coherence decreases both the critical shear stress and the atomic displacement required for phase transformation, thereby promoting the nucleation of FCC phases under external stress perturbations. Consequently, an enhanced propensity for the HCP → FCC transformation is demonstrated in the EB-PBF specimen, as evidenced by the high fraction of the stress-induced FCC phase. These structures are able to improve strength–ductility synergy; the details will be discussed in the following section.

### 4.2. Strengthening Mechanisms

To elucidate the strengthening mechanisms responsible for the pronounced strength enhancement of the L-PBF and EB-PBF specimens, detailed quantitative analyses were conducted for solid-solution strengthening, grain refinement strengthening, and dislocation strengthening:

(1) Solid-solution strengthening

Solid-solution strengthening refers to the mechanism by which solute atoms are incorporated into the solvent lattice, where their elastic interactions with dislocations impede dislocation motion, thereby enhancing the strength of the material. In Ti alloys, solid-solution strengthening is generally regarded as one of the main strengthening mechanisms, and its strength contribution can be calculated as follows [49,50]:

(1) $$\sigma_{\mathrm{SS}\left(\mathrm{Al},\;V\right)}\;=\;{f_{\alpha }\;\times \;(\sum_{i}{B_{i}}^{3/2}X_{i})}^{2/3}\;+\;{f_{\beta }\;\times \;(\sum_{i}{B_{i}}^{3/2}X_{i})}^{2/3}$$

where $B_{i}$ is the strengthening coefficient for the solute *i* and $X_{i}$ is the atomic concentration of the solute *i*. The elemental composition of each specimen is shown in the Supplementary Table S1. The solid-solution strengthening contributions from aluminum and vanadium in the L-PBF and EB-PBF specimens are approximately 128 MPa and 115 MPa, respectively.

Among all kinds of solute elements, oxygen serves as a much stronger solid-solution strengthening element than aluminum or vanadium. Its strengthening contribution in the present alloy can be evaluated using the Labusch model [51], as shown in the following equation:

(2) $$\sigma_{\mathrm{SS}(O)}\;=\;\frac{1}{S_{F}}{\left(\frac{F_{m}^{4}c^{2}w}{4Gb^{9}}\right)}^{1/3}$$

*SF* denotes the Schmid factor, and *F*_m_ is the maximum interaction force between an oxygen solute atom and a dislocation, taken as 6.22 × 10^−10^ N in the present calculation [51]. The parameter *c* represents the atomic fraction of the oxygen solute. The distance between a solute atom and a dislocation is denoted as *w*, which is approximated as 5*b* [52], where *b* is the Burgers vector (0.295 nm in this study [53]). *G* is the shear modulus of the alloy, taken as 44 GPa [54]. The oxygen content and shape factor (*S*_F_) were measured as 0.12 wt.% and 0.31 for the L-PBF specimen and 0.24 wt.% and 0.32 for the EB-PBF specimen, respectively. Since the solubility of interstitial oxygen in HCP is high [55], the solid-solution strengthening of the BCC phase by interstitial oxygen can be ignored. The strengthening contributions from oxygen in the L-PBF and EB-PBF specimens are approximately 151 MPa and 232 MPa, respectively.

(2) Grain boundary strengthening

Grain boundary strengthening, as described by the Hall–Petch relation, enhances the strength of metals by reducing grain size. The underlying mechanism lies in the impediment of dislocation motion by grain boundaries. Due to the crystallographic misorientation and atomic disorder at grain boundaries, dislocations cannot pass directly through; instead, they must activate new dislocation sources within adjacent grains to continue slip. This process increases the critical stress required for plastic deformation, thereby elevating the yield strength. The Hall–Petch relation can be expressed as

(3) $$\sigma_{\mathrm{GB}}\;=k\;\times \left(d_{\left(L/EB-TC4\right)}^{-\frac{1}{2}}-d_{\left(TC4\right)}^{-\frac{1}{2}}\right)$$

where *d* is the average grain size, and *k* is the Hall–Petch constant. For the calculation of L-PBF TC4 alloy, the value of *k* is taken as 0.15 MPa m^1/2^ [56], with *d*_(TC4)_ = 1.2 μm [47]. When calculating for the EB-PBF TC4 specimen, the range of *k* is 0.5–0.7 MN m^−3/2^ [57], *d*_(L-TC4)_ = 0.7 μm, *d*_(E-TC4)_ = 102 μm (Figure S2). In L-PBF specimens, strengthening is primarily controlled by dislocation obstruction within the α′ martensite, making α′ lath thickness the key parameter for boundary strengthening. In contrast, EB-PBF specimens contain a dual-phase (α + β) structure where dislocation motion is hindered by both α lamellae and prior β grain boundaries, necessitating the use of prior β grain size as the relevant strengthening scale. The grain boundary strengthening contributions in the L-PBF and EB-PBF specimens are approximately 42 MPa and 36 MPa, respectively.

(3) Dislocation strengthening

Dislocation strengthening, one of the most important strengthening mechanisms in metallic materials, originates from the elastic interactions between dislocations. During plastic deformation, the dislocation density significantly increased, leading to frequent intersections and the formation of entangled dislocation networks. This increase in dislocation density significantly impedes the glide of mobile dislocations, thereby raising the flow stress—a phenomenon commonly referred to in engineering practice as work hardening or strain hardening. The contribution of dislocation strengthening can be quantitatively estimated using the Taylor model, expressed as

(4) $$\sigma_{D}=M\alpha \mu b\rho^{\frac{1}{2}}$$

where *M* is the Taylor factor, which is 1 for HCP metals, *α* is the dislocation interaction constant (taken as 0.2 for Ti alloys), *μ* is the shear modulus (taken as 44 GPa for Ti alloys), *b* is the Burgers vector (taken as 0.2951 nm for Ti alloys), and *ρ* is the dislocation density [54].

The *ρ* of the as-built specimens can be calculated based on the EBSD results using the following formula:

(5) $$\rho \;=\;\frac{2\theta_{\mathrm{KAM}}}{\mathrm{Xb}}$$

where *X* is the step size, $\theta_{\mathrm{KAM}}$ is the average KAM values, defined as

(6) $$\theta_{\mathrm{KAM}}=\sum f_{i}\theta_{i}$$

where *θ_i_* and *f_i_* are the local misorientation and its frequency, respectively. The corresponding geometrically necessary dislocations (GNDs) of the L-PBF and EB-PBF specimens can be calculated as 1.1 × 10^16^ m^−2^ and 6.2 × 10^15^ m^−2^, respectively. This is consistent with the microstrain calculations, as shown in the Supplementary Figure S3. The dislocation strengthening contributions in the L-PBF and EB-PBF specimens are approximately 274 MPa and 205 MPa, respectively.

In addition, dislocations also significantly influence the plasticity of materials. During the plastic deformation stage, the elongation of the material can be described by Equation (7):

(7) $$e\;=\;b\rho_{m}\bar{x}$$

where $\rho_{m}$ denotes the mobile dislocation density and $\bar{x}$ represents the average dislocation mean free path. As shown in Figure 6a, the EB-PBF specimen exhibits a gradual work-hardening behavior throughout the plastic deformation stage, suggesting that a large fraction of dislocations is mobile. TEM observations revealed an average dislocation spacing of approximately 120 nm. Assuming that 30% of the dislocations are mobile, substitution of these values into Equation (7) yields an estimated plastic strain attributable to dislocation motion of approximately 7%. The remaining elongation is likely attributed to the combined effects of HAGBs, the BCC phase, and the FCC phase. HAGBs are more effective at impeding microcrack propagation and reducing susceptibility to brittle fracture than LAGBs. The BCC phase possesses more active slip systems, making it more prone to plastic deformation. The strain-hardening exponents for the L-PBF and EB-PBF specimens were determined to be 0.077 and 0.092, respectively. This high strain-hardening capacity in the EB-PBF specimen promotes rapid hardening upon localized plastic deformation, thereby effectively suppressing necking and enhancing uniform elongation. Furthermore, the deformed FCC phase also contributes favorably to the material’s ductility. For instance, Ding et al. [58] promoted FCC formation in additively manufactured pure Ti by increasing the oxygen content, enabling the material to overcome the strength–ductility trade-off and achieve a ductility exceeding 20%.

In summary, the additional strength of the EB-PBF specimen arises mainly from solid-solution strengthening, whereas the contribution from grain refinement is comparatively minor. The thick α-laths are expected to reduce the microstrain and extend the dislocation mean free path, thereby promoting homogeneous plastic deformation and enhancing elongation.

(4) FCC Phase Strengthening

It is interesting to find the FCC phase and its twins in the deformed EB-PBF specimens. Because FCC-like Ti phases may be introduced during TEM specimen preparation, particularly by focused ion beam (FIB) irradiation, the TEM foils in this study were prepared by Ar-ion milling [59]. Although FCC in Ti alloys with twinning has been investigated by simulations, the FCC and its twins are difficult to form in HCP-Ti due to its high SFE (300 mJ/m^2^) [60,61,62]. Recent work by Wang et al. [63] has shown that FCC Ti can be synthesized in situ in the L-PBF TC4–0.1 wt.%O alloy through the combined effects of oxygen and thermal cycling. Molecular dynamics (MD) simulations revealed that the formation energy of oxygen in octahedral interstitial sites is lower in the FCC structure than in HCP, thereby promoting the formation of the FCC phase. Their work further indicated that the FCC phase and its twins can provide a strengthening of approximately 128 MPa without compromising ductility.

**Figure 10:**
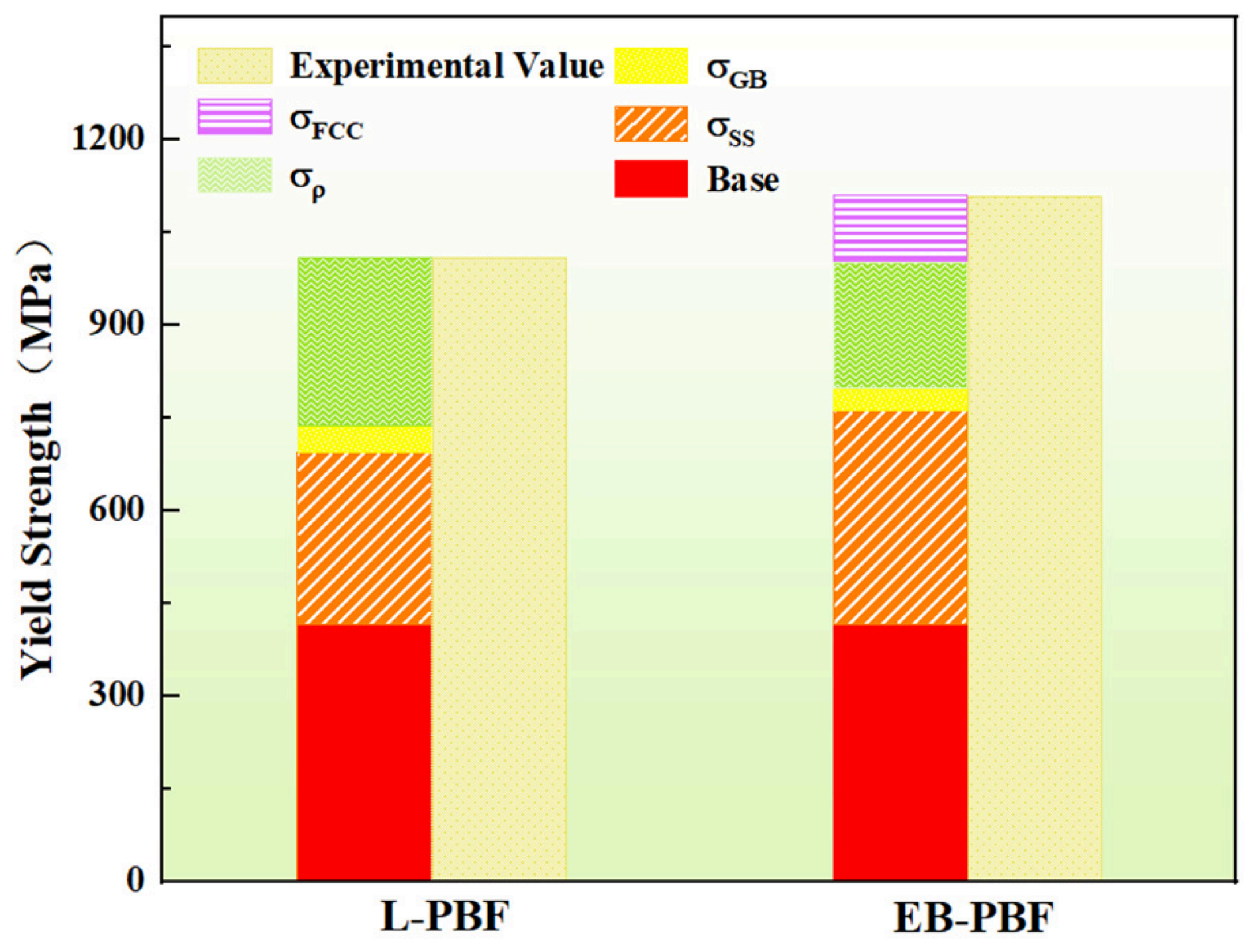
Comparison of the experimental yield strengths with the strengths calculated from the individual strengthening contributions.

Apart from lattice parameters, the deformation-induced FCC phase observed in the EB-PBF specimens in this study is likely associated with the high oxygen content (0.24 wt.%) in the material. The FCC + HCP dual-phase structure and deformation twins enable enhancements in both strength and work-hardening capacity [63,64]. The nanoscale FCC and its twins in the EB-PBF specimen are estimated to contribute a strength increment of approximately 107 MPa. A comparison between the calculated strengths and experimental results of the L-PBF and EB-PBF specimens reveals reasonable agreement. The detailed strength contribution is shown in Figure 10 and the Supplementary Table S4. The high uniform elongation (6.8 ± 0.3%) of the EB-PBF specimen further supports the role of the FCC phase and its twins in promoting coordinated deformation and improved ductility. These findings suggest that the stress-induced formation of the FCC phase in oxygen-enriched EB-PBF specimens can effectively enhance the strength of titanium alloys, offering a practical microstructural design approach for achieving a strength–ductility synergy via additive manufacturing. It should be noted that oxygen was intentionally introduced to evaluate its effect on the microstructure and mechanical properties, and the oxygen content of 0.24 wt.% was measured directly in the as-built EB-PBF specimens. This oxygen level and the associated tensile properties were reproducible for the specimen dimensions investigated in this study, whereas their reproducibility in larger components requires further validation.

## 5. Conclusions

This work systematically compares the microstructure and mechanical properties of the L-PBF and EB-PBF specimens. Based on the experimental results and analysis, the following conclusions can be drawn:

(1) The distinct thermal histories of the two processes result in markedly different microstructures. Rapid cooling during L-PBF promotes the formation of fine acicular α′ martensite, whereas the high preheating temperature and moderate cooling rate of EB-PBF suppress martensitic transformation and favor the formation of a stable lamellar (α + β) structure.
(2) The L-PBF specimens possess high strength and acceptable ductility, with a yield strength of 1016 ± 32 MPa, and an elongation of 10.0 ± 0.9%. In comparison, the EB-PBF specimen achieves a strength–ductility synergy, with a yield strength of 1120 ± 12 MPa and an elongation of 11.1 ± 1.3%.
(3) The synergistic strengthening observed in the EB-PBF specimens arises primarily from the processing features and high oxygen content (0.24 wt.%). Oxygen contributes to solid-solution strengthening and may promote the formation of deformation-induced FCC-structured regions, while the lamellar microstructure facilitates crack deflection and helps retain ductility.

## Data Availability

The original contributions presented in this study are included in the article/Supplementary Material. Further inquiries can be directed to the corresponding author.

## Supplementary Materials

The following supporting information can be downloaded at https://www.mdpi.com/article/10.3390/ma19153300/s1, Figure S1. True stress-strain curves and work hardening rate curves of the L-PBF and EB-PBF specimens; Figure S2. BSE image for prior-β grains of the EB-PBF specimen; Figure S3. Linear relationship between peak broadening δ·cos*θ* and 2sin*θ*; Table S1. Elemental content in L-PBF and EB-PBF specimens; Table S2. Microstrain calculation-related data for L-PBF specimens; Table S3. Microstrain calculation-related data for EB-PBF specimens; Table S4 Summary of strengthening mechanism contributions for L-PBF and EB-PBF specimens. References [49,50,51,52,53,54,56,57,65] are cited in the Supplementary Materials.
