# Supplementary material for: A Comparative Study on Microstructure and Mechanical Properties of Ti-6Al-4V Fabricated by Laser/Electron Beam Powder Bed Fusion
Source: Materials (Basel). 2026 Aug 4;19(15):3300. doi: 10.3390/ma19153300 (PMC13468040; doi:10.3390/ma19153300)
Supplement: Supplementary file 1 [file materials-19-03300-s001.zip › materials-4430919-supplementary.pdf]

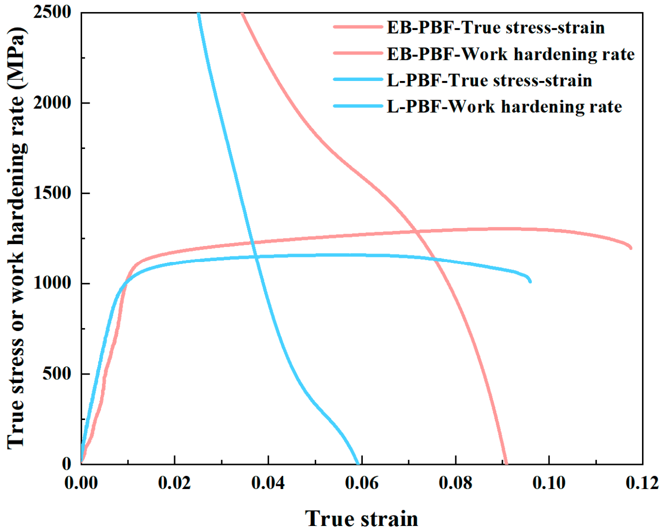

Supplementary Figure S1. True stress-strain curves and work hardening rate curves of the L-PBF and EB-PBF specimens

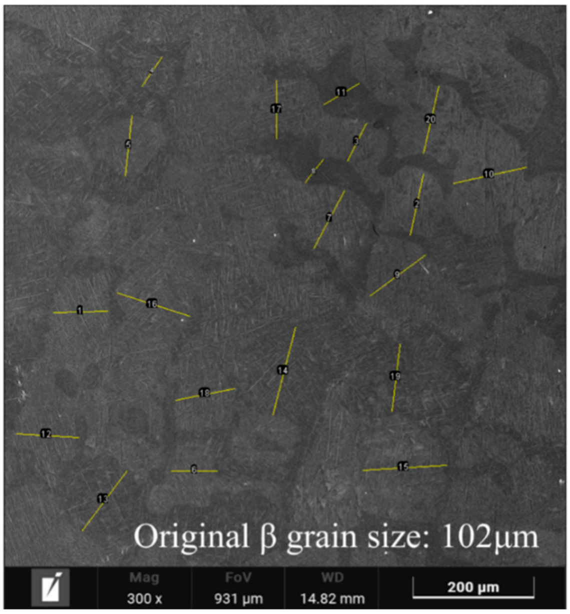

Supplementary Figure S2. BSE image for prior-β grains of the EB-PBF specimen.

According to the approach proposed by Williamson and Hall, the broadening of XRD peaks primarily originates from two contributions: crystallite size ($\delta_{(D,\ hkl)}$) and microstrain ($\delta_{(\varepsilon,\ hkl)}$) [65]. In the present study, the average grain size of the as-printed alloy, as determined by BSE, ranges from approximately 0.7 to 1.9 μm. At this scale,

peak broadening due to crystallite size effects can be negligible. The observed diffraction peak broadening is primarily attributed to microstrain, which can be quantified using Eqs. S1-S3:

$$\delta_{hkl} = \delta_{(D,\ hkl)} + \delta_{(\varepsilon,\ hkl)} \quad \text{(S1)}$$

$$\delta_{hkl} \cdot \cos\theta_{hkl} \approx 2\varepsilon \cdot \sin\theta_{hkl} \quad \text{(S2)}$$

$$\delta_{hkl} = \sqrt{\delta^2_{hkl(\text{as-built})} - \delta^2_{hkl(\text{powder})}} \quad \text{(S3)}$$

$\delta_{hkl(\text{as-built})}$ and $\delta_{hkl(\text{power})}$ are the half-height widths of the XRD peaks of the printed alloy and the original powder, respectively. The powder specimen was used as a reference without strain. Linear fitting of Eq. S2 can yield micro-strain, as shown in supplementary Fig. S3 (the details are given in the Supplementary Tables S2 and S3):

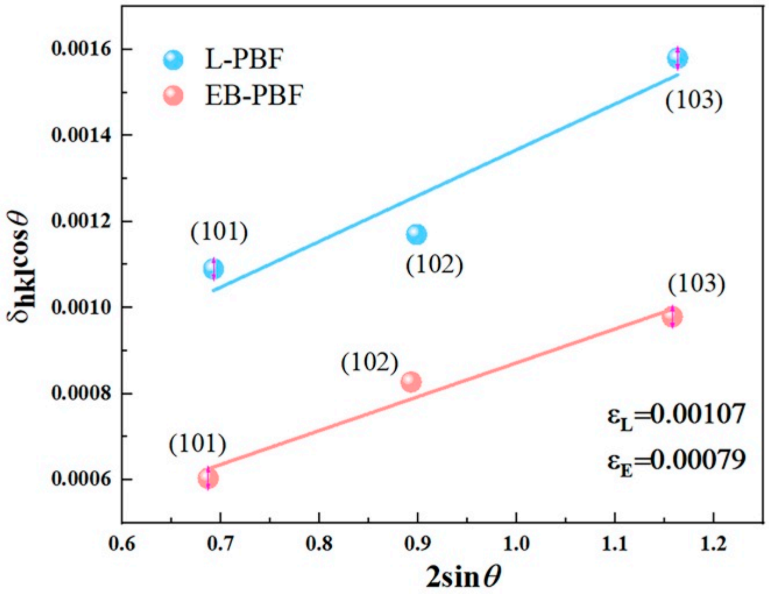

Supplementary Figure S3. Linear relationship between peak broadening $\delta \cdot \cos\theta$ and $2\sin\theta$.

Supplementary Table S1 Elemental content in L-PBF and EB-PBF specimens

	**Ti (at.%)**	**Al (at.%)**	**V (at.%)**	**O (at.%)**
L-PBF-HCP	87.81	7.68	4.16	0.35
E-PBF-HCP	86.30	10.38	2.64	0.68
E-PBF-BCC	86.07	8.64	5.29	-

Supplementary Table S2 Microstrain calculation-related data for L-PBF specimens

	L-PBF $_{as\text{-}built}$	L-PBF $_{powder}$	L-PBF	
2θ	$\delta_{hkl\ (as\text{-}built)}$	$\Delta_{hkl(powder)}$	$\delta_{hkl}\cdot\cos\theta$	$2\sin\theta$
40.5233	0.46609	0.29526	0.00109	0.692615626
53.38468	0.46088	0.35217	0.001165	0.898399116
71.1227	0.54779	0.45037	0.001583	1.163148689

Supplementary Table S3 Microstrain calculation-related data for EB-PBF specimens

	EB-PBF $_{as\text{-}built}$	EB-PBF $_{powder}$	EB-PBF	
2θ	$\delta_{hkl\ (as\text{-}built)}$	$\Delta_{hkl(powder)}$	$\delta_{hkl}\cdot\cos\theta$	$2\sin\theta$
40.19304	0.30286	0.22641	0.000603	0.687205
53.03171	0.33757	0.26244	0.000827	0.892891
70.74136	0.39964	0.3495	0.000979	1.157728

Supplementary Table S4 Summary of strengthening mechanism contributions for L-PBF and EB-PBF specimens

Strengthening Type	Key Assumptions	L-PBF (MPa)	EB-PBF (MPa)
Al+V Solid-solution [49, 50]	B_i coefficients, atomic fraction of alloy elements	~128	~115
Oxygen Solid-solution [51-54]	F_m=6.22×10^{-10} N, w=5b, b=0.295, G=44 GPa	~151	~232
Grain boundary [56, 57] (Hall-Petch)	k=0.15 MPa m$^{1/2}$ (L-PBF), k=0.5-0.7 MN·m$^{-3/2}$ (EB-PBF)	~42	~36
Dislocation strengthening [54] (Taylor)	M=1, α=0.2, b=0.295 nm	~274	~205
FCC phase strengthening	Strengthening value obtained by subtracting the sum of all other contributions from the measured yield strength	-	~107

Reference

[49] Zhao, G.H.; Liang, X.Z.; Kim, B.; Rivera-Díaz-del-Castillo, P.E.J. Modelling strengthening mechanisms in beta-type Ti alloys. Mater. Sci. Eng. A 2019, 756, 156–160. https://doi.org/10.1016/j.msea.2019.04.027.
[50] Zhang, T.; Zhu, J.; Yang, T.; Luan, J.; Kong, H.; Liu, W.; Cao, B.; Wu, S.; Wang, D.; Wang, Y.; et al. A new α + β Ti-alloy with refined microstructures and enhanced mechanical properties in the as-cast state. Scr. Mater. 2022, 207, 114260. https://doi.org/10.1016/j.scriptamat.2021.114260.
[51] Kariya, S.; Fukuo, M.; Umeda, J.; Kondoh, K. Quantitative Analysis on Light Elements Solution Strengthening in Pure Titanium Sintered Materials by Labusch Model Using Experimental Data. Mater. Trans. 2019, 60(2), 263–268. https://doi.org/10.2320/matertrans.Y-M2018849.
[52] Otsu, A.; Doi, A.; Onda, T.; Kimura, T.; Nakamoto, T.; Chen, Z.-C. Microstructure and enhanced strength and ductility of Ti-Zr-O alloys prepared by a laser powder bed fusion process. Addit. Manuf. 2024, 85, 104170. https://doi.org/10.1016/j.addma.2024.104170.
[53] Chen, K.; Li, H.; Huang, D.J.; Shen, X.; Jia, N. Avoiding oxygen-induced early fracture in titanium with high strength via entangled grains through laser powder bed fusion. Scr. Mater. 2023, 222, 115051. https://doi.org/10.1016/j.scriptamat.2022.115051.
[54] Zhang, C.; Bao, X.; Hao, M.; Chen, W.; Zhang, D.; Wang, D.; Zhang, J.; Liu, G.; Sun, J. Hierarchical nano-martensite-engineered a low-cost ultra-strong and ductile titanium alloy. Nat. Commun. 2022, 13, 5966. https://doi.org/10.1038/s41467-022-33710-1.
[56] Fu, Y.; Yu, M.; Wu, D.; Wang, Z.; Tong, J.; Wang, D.; Guo, N. Microstructure modulation and property enhancement via an underwater induction heating treatment strategy for underwater laser-directed energy deposition of Ti-6Al-4V. Virtual Phys. Prototyp. 2024, 19(1), e2368659. https://doi.org/10.1080/17452759.2024.2368659.
[57] Banerjee, D.; Williams, J.C. Perspectives on Titanium Science and Technology. Acta Mater. 2013, 61(3), 844–879. https://doi.org/10.1016/j.actamat.2012.10.043.
[65] Williamson, G.K.; Hall, W.H. X-ray line broadening from filed aluminium and wolfram. Acta Metall. 1953, 1(1), 22–31. https://doi.org/10.1016/0001-6160(53)90006-6
